# The effect of endoscopic transsphenoidal somatotroph tumors resection on pituitary hormones: systematic review and meta-analysis

**DOI:** 10.1186/s12957-023-02958-2

**Published:** 2023-03-01

**Authors:** Ding Nie, Qiuyue Fang, Wakam Wong, Songbai Gui, Peng Zhao, Chuzhong Li, Yazhuo Zhang

**Affiliations:** 1 Beijing Neurosurgical Institute, Capital Medical University, Beijing, China; 2 Department of Neurosurgery, Beijing Tiantan Hospital, Capital Medical University, Beijing, China

**Keywords:** Endoscopic transsphenoidal, Pituitary neuroendocrine tumors (PitNETs), Pituitary function

## Abstract

**Purpose:**

Currently, endoscopic transsphenoidal surgery is the main treatment for pituitary neuroendocrine tumors (PitNETs). Excision of the tumor may have positive or negative effects on pituitary endocrine function, and the pituitary function of somatotroph tumors is a point of particular concern after the operation. This study aimed to conduct a meta-analysis on the effect of endoscopic transsphenoidal somatotroph tumor resection on pituitary function.

**Methods:**

A systematic literature search was conducted for articles that included the evaluation of pituitary target gland before and after endoscopic transsphenoidal pituitary tumor resection and were published between 1992 and 2022 in PubMed, Cochrane, and Ovid MEDLINE.

**Results:**

Sixty-eight studies that included biochemical remission rates in 4524 somatotroph tumors were concluded. According to the 2000 consensus, the biochemical remission rate after transsphenoidal endoscopic surgery was 66.4% (95% *CI*, 0.622–0.703; *P* = 0.000), the biochemical remission rate was 56.2% according to the 2010 consensus (95% *CI*, 0.503–0.620; *P* = 0.041), and with the rate of biochemical remission ranging from 30.0 to 91.7% with investigator’s definition. After endoscopic resection, adrenal axis dysfunction was slightly higher than that before surgery, but the difference was not statistically significant. Hypothyroidism was 0.712 times higher risk than that before surgery (*OR* = 0.712; 95% *CI*, 0.527–0.961; *P* = 0.027). Hypogonadism was 0.541 times higher risk than that before surgery (*OR* = 0.541; 95% *CI*, 0.393–0.746; *P* = 0.000). Hyperprolactinemia was 0.131 times higher risk than that before surgery (*OR* = 0.131; 95% *CI*, 0.022–0.783; *P* = 0.026). The incidence of pituitary insufficiency was 1.344 times the risk before surgery after endoscopic resection of somatotroph tumors, but the difference was not statistically significant.

**Conclusions:**

In patients with somatotroph tumors after undergoing endoscopic surgery, the risk of dysfunction and pituitary insufficiency tend to increase, while preoperative thyroid insufficiency, gonadal insufficiency, and hyperprolactinemia will be partially relieved.

**Supplementary Information:**

The online version contains supplementary material available at 10.1186/s12957-023-02958-2.

## Introduction

Pituitary neuroendocrine tumor (PitNET) is the third most common intracranial tumor which can be divided into functional and non-functional according to clinical manifestations and histological features [1]. Somatotroph tumors, characterized by excessive secretion of growth hormone (GH), are a kind of functional tumor, accounting for about one-fifth of PitNETs [2]. Continuous GH excess can lead to acromegaly and increase the incidence of systemic diseases such as cardiovascular disease, digestive disease, and endocrine disease so the quality of life and life expectancy are often affected [3, 4]. At present, the main treatment is endoscopic transsphenoidal resection of tumors, but the biochemical remission rate after surgery is about 50–60% [5, 6]. Although endoscopic transsphenoidal surgery has the advantages of visualization and minimal invasion, the surgery itself often has positive or negative effects on pituitary function, which is not only limited to the changes of growth hormone and IGF-1 but also includes thyroid, adrenal, and gonadal axis hormones, which need drug substitution therapy [7]. Changes in pituitary hormone require clinicians to manage patients with long-term follow-up after surgical resection of the tumor and choose radiotherapy and drug therapy if necessary. The primary objective of this meta-analysis was to assess pituitary target function before and after endoscopic transnasal resection of somatotroph tumor and to provide evidence-based findings for the rational development of postoperative treatment strategies to achieve biochemical remission and the need for alternative hormone therapy. At the same time, a more specific endocrine remission rate was shown.

## Methods

### Search strategy

This meta-analysis was performed by the Preferred Reporting Items for Systematic Reviews and Meta-Analyses (PRISMA) guidelines. Two independent investigators (Nie and Fang) conducted literature (from inception to May 15, 2022), using the following databases: PubMed, Cochrane, and Ovid MEDLINE. Search strategy based on keywords is as follows: “pituitary adenoma,” “growth hormone pituitary adenoma,” “growth hormone-producing pituitary adenoma,” “somatotroph tumor,” “acromegaly,” “endoscopic surgery,” “endoscopic transsphenoidal surgery,” “anterior pituitary function,” “pituitary insufficiency,” “hypothyroidism,” “thyroid insufficiency,” “adrenal insufficiency,” “hypoadrenalism,” and “endoscopic.”

Two researchers (Nie and Fang) independently conducted literature screening, data extraction, and quality evaluation. If there is any disagreement, another researcher will help mediate it (Zhang). The inclusion criteria were English original research articles. Case reports, conference abstracts, meta-analyses, and reviews were excluded. This article should describe the pituitary hormone before and after endoscopic transsphenoidal pituitary tumor surgery in patients with somatotroph tumors. References to all selected articles are reviewed.

### Data extraction

Two reviewers (Nie and Fang) independently extracted the data from each of the studies selected. We extracted continuous biochemical remission results for analyzing biochemical remission rates and extracted number analysis for each target axis.

### Statistical analysis

Meta-analysis was performed using CMA v 3.0. For categorical data, the odds ratio (OR) was used for effect-scale consolidation. All effect sizes were expressed with 95% confidence intervals (CI). We measured heterogeneity by the chi-squared Cochran’s *Q*-test and *I*^2^ statistics. If *Q* reaches a *P* < 0.1 or *I*^2^ > 50%, there is a significant heterogeneity among studies. The random-effect model was selected for analysis. Meta-regression was used to explore if the following priori-defined covariates accounted for heterogeneity of study-level effect sizes. Each of these variables was used to assess the association with the outcome through univariate analysis. Sensitivity analyses were conducted, and no significant sensitivity from all studies was detected. Publication bias was tested via a funnel plot.

Quality of included studies is according to Nottingham-Ottawa scale. In meta-regression, study quality was not a significant determinant of the biochemical remission rate. Twenty-three studies were using the 2000 consensus (*R*^2^ = 0; *P* = 0.53) and 28 studies using the 2010 consensus (*R*^2^ = 0; *P* = 0.87).

## Results

### Literature retrieval results

**Fig. 1:**
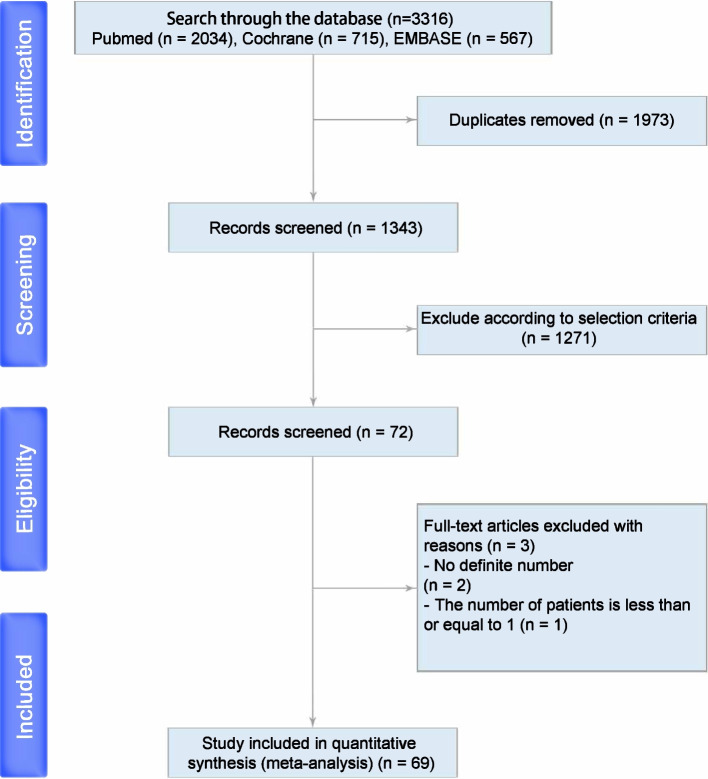
PRISMA 2009 flow diagram for identification of papers included in the systematic review and meta-analysis

Preliminary screening found 3316 articles. After reading the title, abstract, and full text, exclude literature that does not meet the inclusion criteria. The final study included 69 studies with a total of 4635 patients undergoing the same surgical procedure, endoscopic transsphenoidal surgery (Supplementary Table 1). The literature screening flow chart and results are shown in Fig. 1.

### Evaluation of biochemical remission rate

**Fig. 2:**
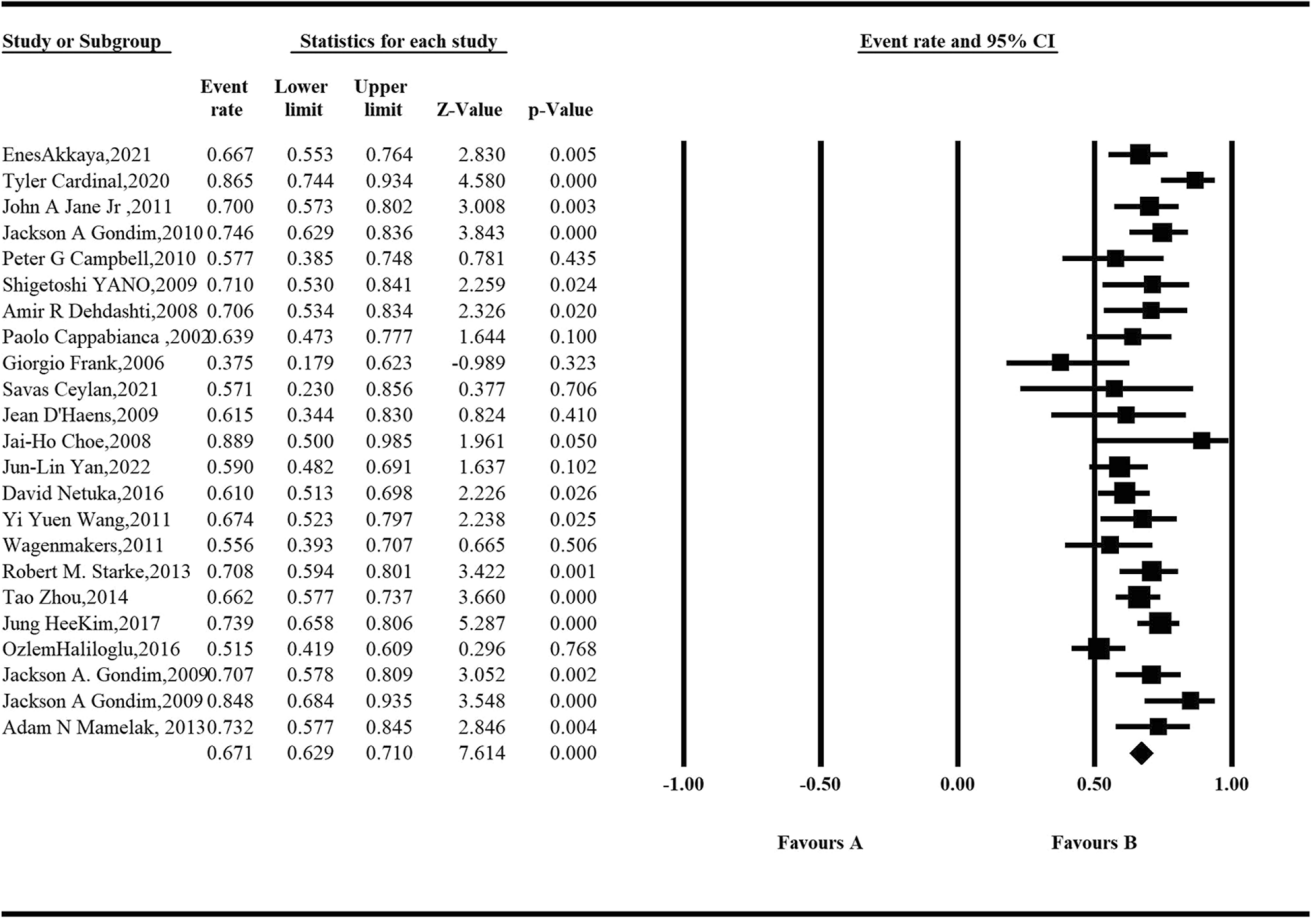
The biochemical remission rate of somatotroph tumors after endoscopic transsphenoidal surgery according to the meta-analysis (2000 consensus)

**Fig. 3:**
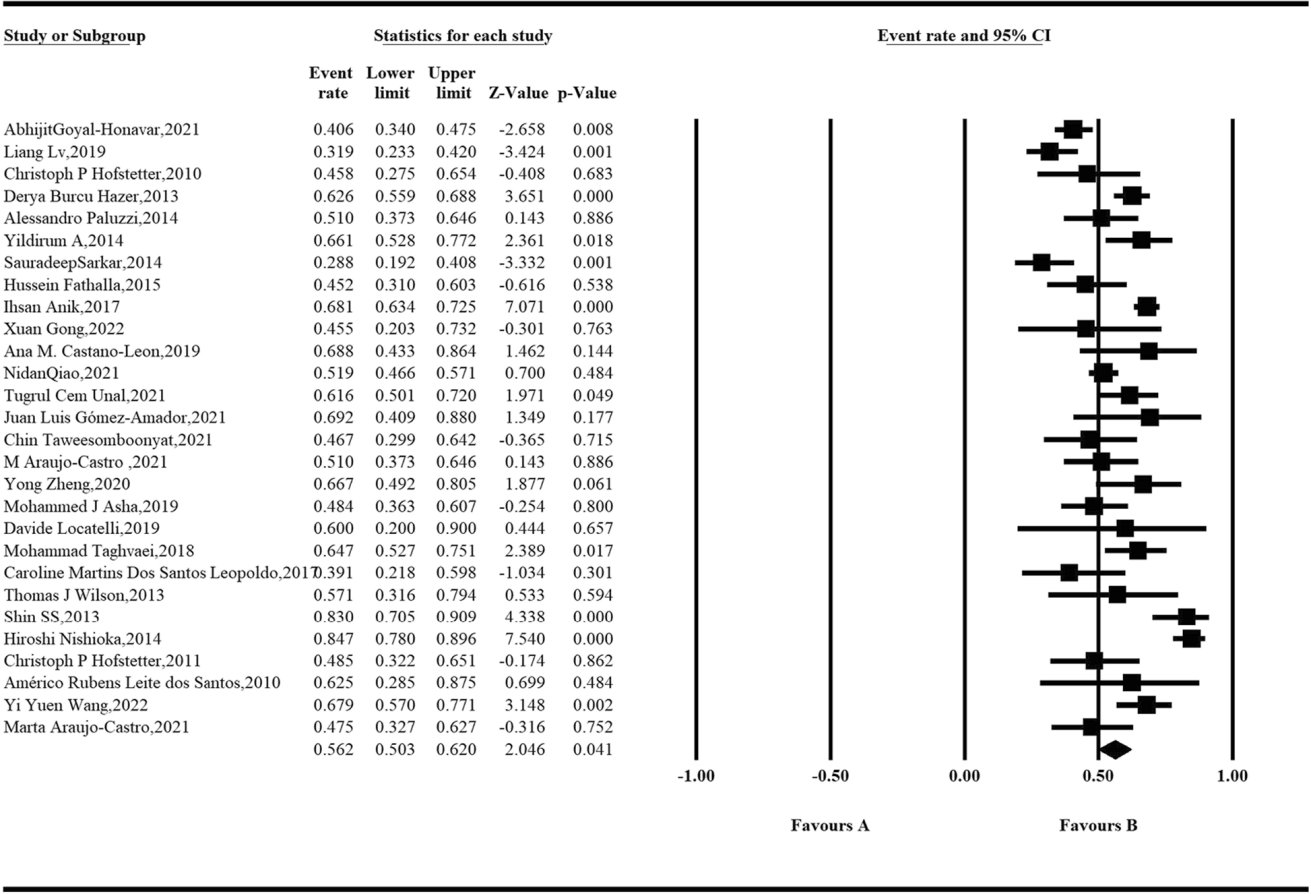
The biochemical remission rate of somatotroph tumors after endoscopic transsphenoidal surgery according to the meta-analysis (2010 consensus)

Sixty-eight studies that included biochemical remission rates in 4018 somatotroph tumors were concluded using the random-effect model. Analysis showed that among the 23 studies according to the 2000 consensus, the pooled rate of biochemical remission was 67.1% and was statistically significant (95% *CI*, 0.629–0.710; *P* < 0.001) (Fig. 2). The heterogeneity of our results was medium (*I*^2^ = 51.018%). To identify the source of heterogeneity in the biochemical remission rate, we conducted a meta-regression to examine the proportion of variance that could be explained by factors. The following variables accounted for *R*^2^: national and center. Among the 28 studies according to the 2010 consensus, the pooled rate of biochemical remission was 56.2% and was statistically significant (95% *CI*, 0.503–0.620; *P* = 0.041) (Fig. 3). The heterogeneity of our results was high (*I*^2^ = 83.735). To identify the source of heterogeneity in the biochemical remission rate, we conducted a meta-regression to examine the proportion of variance that could be explained by factors. The following variables accounted for *R*^2^: country and published year. In addition, we reviewed 16 studies using the investigator’s definition as criteria, with the rate of biochemical remission ranging from 30.0 to 91.7% (Table 1).

**Table 1:** Biochemical remission rate of somatotroph tumors after endoscopic transsphenoidal surgery (investigator’s definition as criteria)

Study or subgroup	Biochemical remission (*n*)	Total (*n*)	Rate	Definition of remission
M. S. Kabil, 2005	41	48	0.854	N/A
A. Rudnik, 2005	10	12	0.833	N/A
W. M. Lui, 2001	5	5	0.917	*GH* ≤ 2 μg/l
Christa C. van Bunderen, 2013	9	30	0.3	Normal IGF-1,
				No active clinical symptoms
Abtin Tabaee, 2009	5	6	0.833	N/A
Guan Sun, 2017	6	7	0.857	Normal GH
Alexander P. Kelly, 2022	1	2	0.5	Normal IGF-I
Chao Tao, 2021	10	13	0.769	2010
Heping Zhou, 2017	67	86	0.779	N/A
Shigetoshi Yano, 2017	26	47	0.553	Inconsistent
J. Lenzi, 2015	15	22	0.682	N/A
Fuyu Wang, 2015	119	180	0.661	N/A
Carlos Takahiro Chone, 2014	6	7	0.857	N/A
P. Cappabianca, 1999	4	5	0.8	N/A
A. Gamea, 1994	4	5	0.8	*GH 5* ≤ ng/ml
Andreja Maric, 2012	15	21	0.714	*IGF-1* < 420 ng/mL and *GH* < 5.0 ng/mL

### Evaluation of anterior pituitary function

**Fig. 4:**
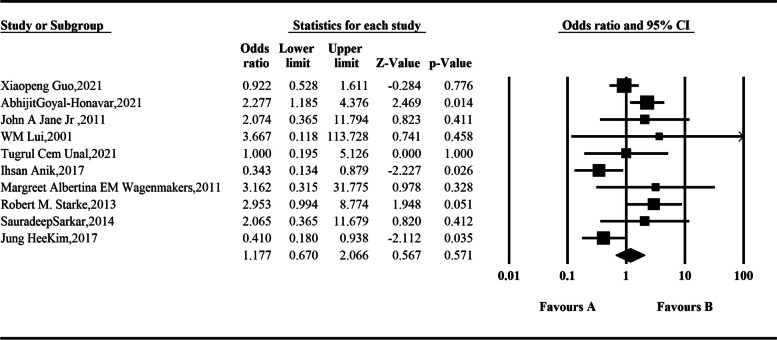
Comparison of adrenal insufficiency before and after operation according to the meta-analysis

This meta-analysis included 10 studies with complete preoperative and postoperative evaluation data of adrenal insufficiency, involving a total of 1566 patients. The random-effect model was used for integration, and the analysis results showed that the proportion of somatotroph tumors with adrenal axis dysfunction after endoscopic resection was slightly higher than that before surgery, but the difference was not statistically significant: *OR* = 1.177 (95% *CI*, 0.670–2.066; *P* = 0.571) (Fig. 4).

**Fig. 5:**
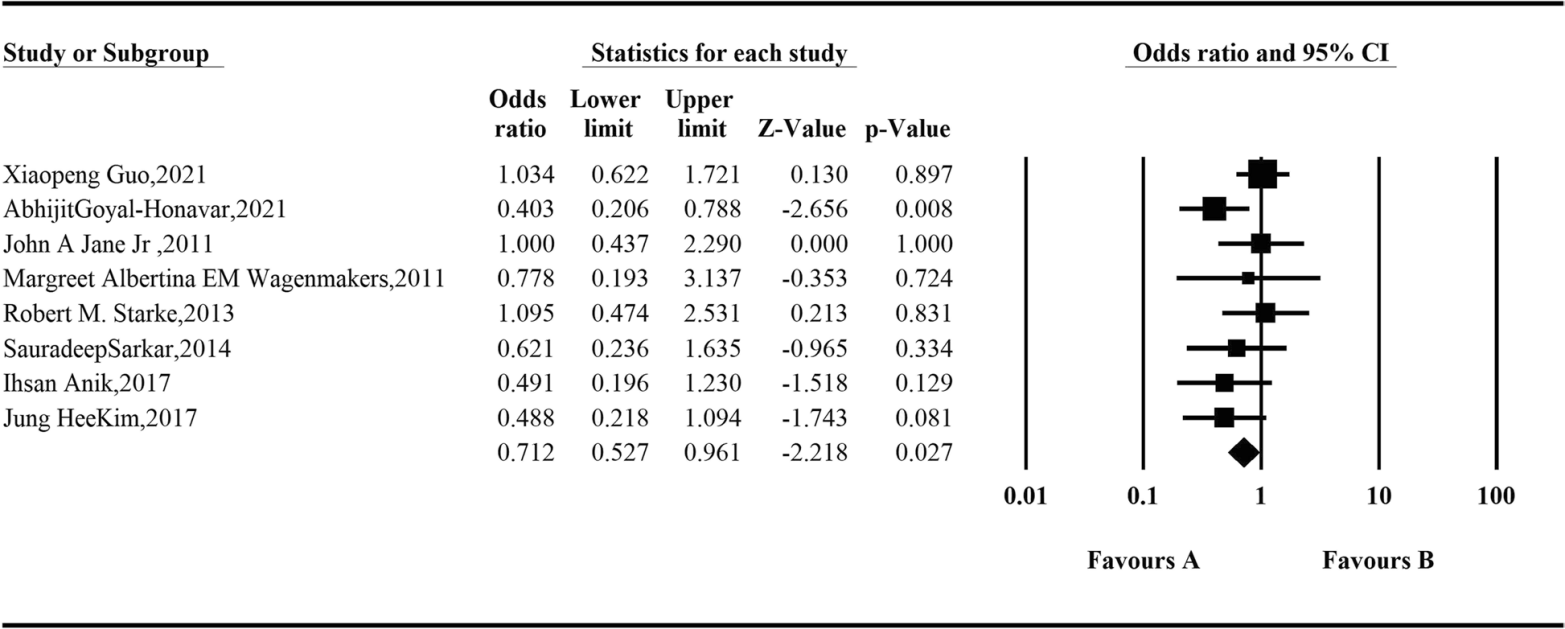
Comparison of hypothyroidism before and after operation according to the meta-analysis

In this meta-analysis, 8 studies with preoperative and postoperative data on hypothyroidism were included. One-thousand four-hundred seventy-three patients were involved. The random-effect model was used for integration, and the analysis results showed that the incidence of hypothyroidism after endoscopic resection of somatotroph tumors was 0.712 times higher than that before surgery, and the difference was statistically significant: *OR* = 0.712 (95% *CI*, 0.527–0.961; *P* = 0.027) (Fig. 5).

**Fig. 6:**
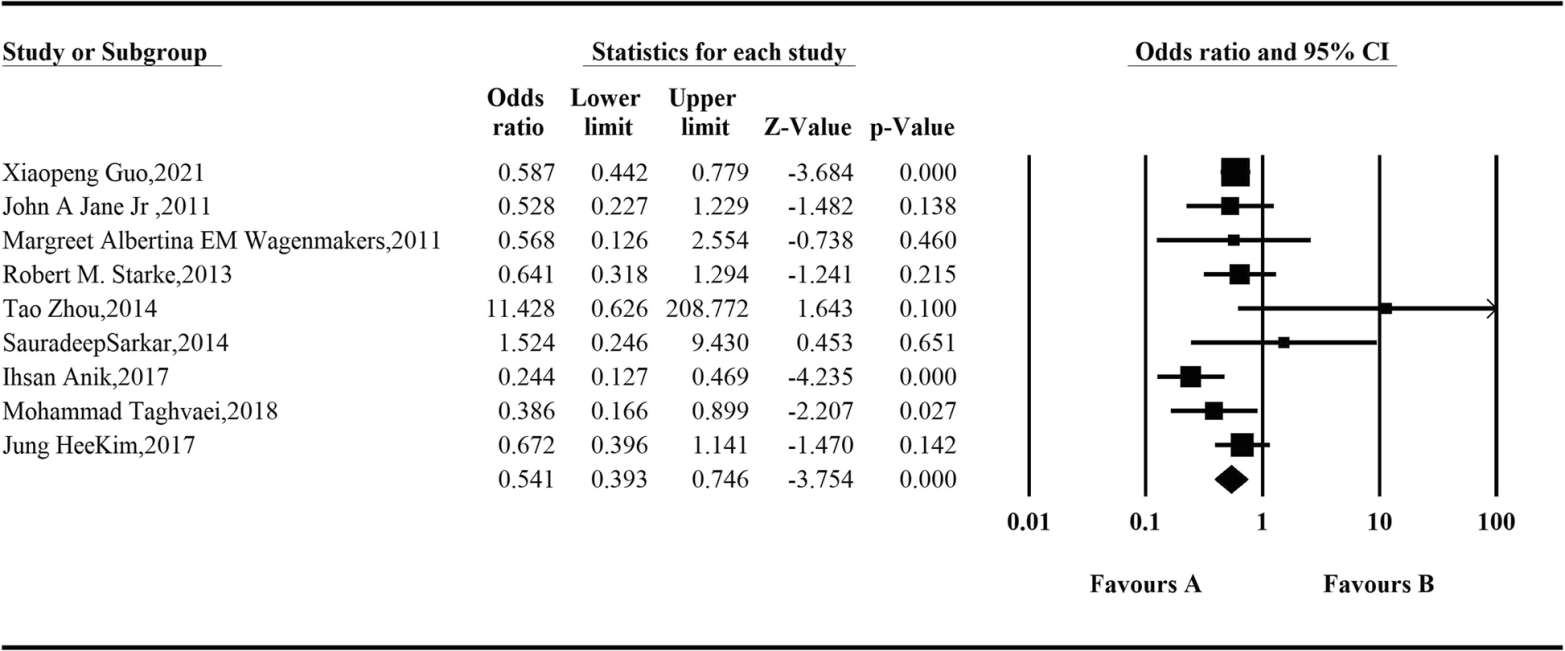
Comparison of hypogonadism before and after operation according to the meta-analysis

Nine studies that assessed hypogonadism data pre- and postoperatively were included. Involved 1488 patients. The random-effect model was used for integration, and the analysis results showed that the incidence of hypogonadism after endoscopic resection of somatotroph tumors was 0.541 times higher than that before surgery, and the difference was statistically significant: *OR* = 0.541 (95% *CI*, 0.393–0.746; *P* < 0.001) (Fig. 6).

**Fig. 7:**
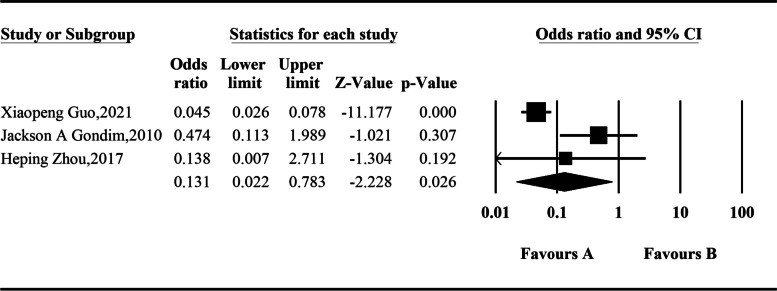
Comparison of hyperprolactinemia before and after operation according to the meta-analysis

Three studies involving 675 patients with hyperprolactinemia were included. The random-effects model was used for integration, and the analysis results showed that the incidence of hyperprolactinemia in endoscopic resection of somatotroph tumors was 0.131 times higher than that before surgery, and the difference was statistically significant: *OR* = 0.131 (95% *CI*, 0.022–0.783; *P* = 0.026) (Fig. 7).

**Fig. 8:**
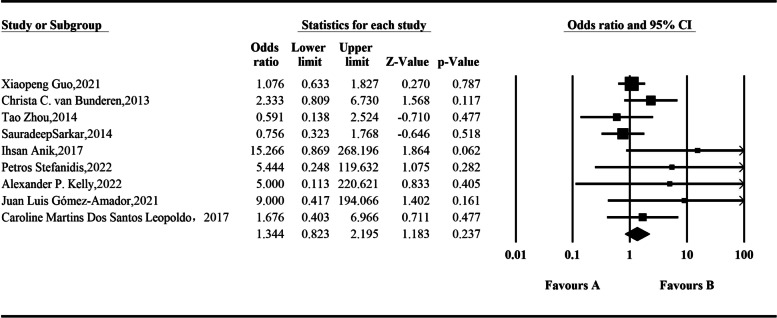
Comparison of pituitary insufficiency before and after operation according to the meta-analysis

A total of 9 studies were included, including 1234 patients with pituitary insufficiency. In integration using a random-effect model, analysis results showed that the incidence of pituitary insufficiency was 1.344 times before surgery after endoscopic resection of somatotroph tumors, but the difference was not statistically significant: *OR* = 1.344 (95% *CI*, 0.823–2.195; *P* = 0.237) (Fig. 8).

### Publication bias evaluation

**Fig. 9:**
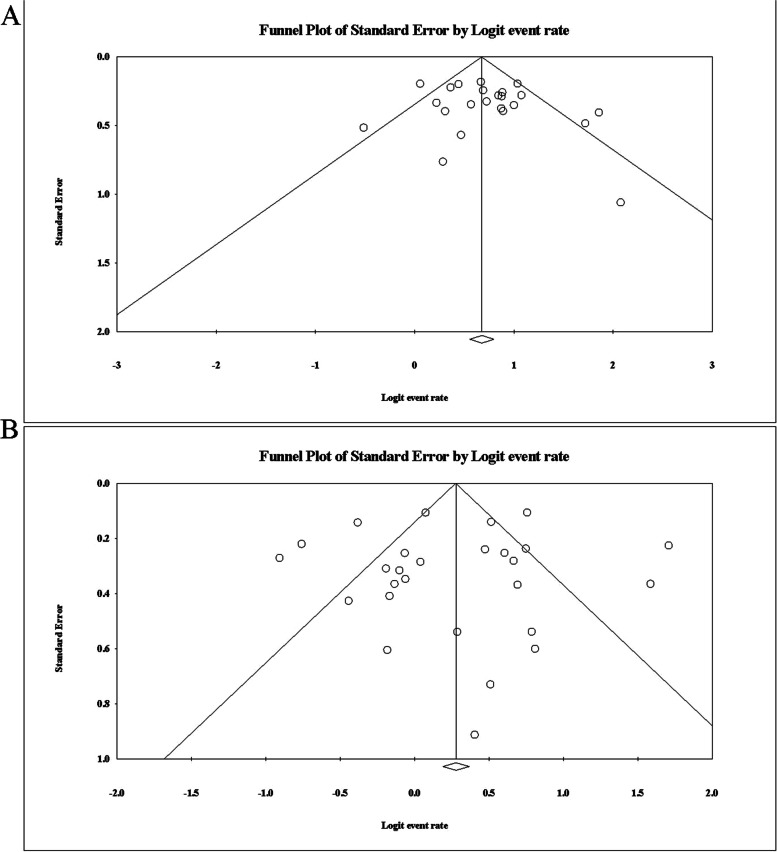
Funnel plot of evaluation of biochemical remission rate. **A** Twenty-three studies using the 2000 consensus. **B** Twenty-eight studies using the 2010 consensus

A funnel plot was used to evaluate the publication bias of studies included in this meta-analysis. The shape of the funnel plot was symmetrical (Fig. 9).

## Discussion

Adenohypophysial neoplasms have recently been renamed pituitary neuroendocrine neoplasms (PitNETs), which are classified as clinically functioning and non-functioning (NF) [8]. Somatotroph tumors are the common subtype of functional PitNETs. Somatotroph tumors are manifested by excessive growth hormone in the body and may eventually lead to acromegaly and other systemic diseases which seriously affect human health [9, 10]. Thus, biochemical remission is the ultimate goal of treatment for somatotroph tumors, not just tumor resection [11]. Since the twenty-first century, with the rapid development of neuroendoscopy, endoscopic transsphenoidal pituitary surgery has become a first-line surgical method for acromegaly patients [12]. To our knowledge, most studies have been conducted to compare the results of microscopic versus endoscopic surgery, and there are no conclusive reports on biochemical response rates after endoscopic treatment of such tumors [13]. We reviewed the literature from 1992 to 2022 to systematically evaluate studies from an international cohort. At the same time, we also summarized the influence of endoscopic surgery on the pituitary function of somatotroph tumors patients, to guide future treatment.

In previous reports, endoscopic surgery did not appear to significantly improve remission rates compared to microscopic surgery [14]. This may be because the advantages of endoscopy lie in the visualization of the surgical field and the small damage to the surgical pathway, while the biological characteristics of the tumor are not changed by the surgical method, which are exactly important factors affecting the remission rate, such as tumor size, invasiveness, secretory activity, and the neuropathological findings [15, 16]. Robust evidence showed that small tumors (< 1 cm) can achieve a good remission rate regardless of the surgical method, and multiple surgeries will create difficulties [11, 17–19]. This is consistent with our observations, and the most important reason is those small tumors are more likely to be completely removed during surgery, resulting in the loss of secretory tumor cells. For large tumors, some researchers have reported a higher tumor removal rate and a higher biochemical response rate with endoscopic transsphenoidal surgery compared with transsphenoidal surgery under the microscope [13, 20]. In some current studies, Knosp or Hardy grading is a better predictor of postoperative remission than tumor size [14, 21, 22]. For example, Yano et al. reported a response rate of 76.9% for Knosp grades 0–2 tumors during endoscopic surgery, compared with 40% for Knosp grades 3–4 tumors [22]. Campbell et al. reported lower biochemical response rates in Hardy grades 3 and 4 tumors [16]. A reasonable explanation is that the tumor with a high grade has a low resection rate and is prone to residual or recurrence, so the remission rate is lower than that of the tumor with a low grade. However, tumors with high-grade grades or large sizes seem to be more likely to be completely resected during endoscopic surgery. For example, endoscopic surgery improves the resection rate of tumors invading the cavernous sinus and improves the biochemical remission rate [23]. Histological invasion is also a major competitor to hormonal remissions, such as both dural invasion, and cavernous sinus invasion can reduce the overall remission rate, but when excision of the capsule is performed, the remission rate is improved [15, 24, 25]. As with microscope surgery, preoperative growth hormone levels and IGF-1 levels are other important predictors of postoperative biochemical remission in endoscopic surgery, that is, preoperative GH and IGF-1 levels are often negatively correlated with surgical results [26–29]. Several other factors have been reported as predictors of remission rates. First, Akkaya et al. reported a lower probability of surgical remission of acromegaly in the T2 hyper signal group, and Alimohamadi et al. reported moderate to enhanced diffusion on DW imaging (DWI) predicted a higher excision rate [30]. Second, the use of neuronavigation or intraoperative hormone monitoring can improve surgical outcomes [31]. Luca D'Angelo et al. showed that stereotactic combined with endoscopic surgery is less invasive [32]. Transsphenoidal endoscopic surgery by a senior surgeon and increased surgical experience are important for improving remission rates [33]. In a prospective study of 50 patients, 28 of 33 functional adenomas achieved a response, and it was proposed that resection via extra-pseudo capsular or intra-pseudo capsular dissection could help improve resection and response rates [34]. Age and sex are predictive of postoperative remission rates in some studies [35–37]. But their results were inconsistent, and more research is needed. Bhawani et al. have suggested that shaving a thin layer of normal pituitary gland along the lumen increases the chances of a tumor being cured [38]. This, too, needs further exploration.

In addition to the postoperative remission rate of somatotroph tumors, we should also pay attention to the functional changes of other hormone axes, because hypopituitarism is one of the common complications after endoscopic transsphenoidal pituitary tumors resection, which need long and careful postoperative management required [39]. Recent studies by Mendel et al. have shown that young age and functional tumors predict new hypopituitarism [40]. However, most reports focus more on the characteristics of tumor growth and surgical techniques which include pituitary gland and stalk manipulation during the operation [15]. As previously mentioned, larger tumor size, higher grade, pituitary apoplexy, multiple surgeries, and invasion of the dura or cavernous sinus are all risk factors for new pituitary dysfunction [41–43]. Compared with a microscope, the use of an endoscope may preserve pituitary function better with a similar degree of resection [44]. Experienced neurosurgeons can better distinguish between tumor pseudocapsule and normal pituitary/stalk during surgery, which is beneficial to protecting endocrine function [45]. Xin Qu et al. also proposed that a pseudocapsule-based extracapsular resection method could improve postoperative outcomes and maintain normal pituitary function [46]. Excessive excision during surgery or thermal damage due to bipolar coagulation in the saddle cavity can lead to anterior lobe dysfunction [47]. Postoperative sellar hematoma is a risk factor for new endocrine dysfunction, and timely removal of hematoma can be partially alleviated [48]. Intraoperative cerebrospinal fluid leakage also increases the incidence of hypopituitarism [49]. The preoperative hypophysial defect is thought to be due to compression and destruction of the normal hypophysial gland by an enlarged mass and may also be due to focal necrosis following compression of the portal circulation [12]. In the literature we collected, the incidence of preoperative adrenal insufficiency was between 2.5 and 6.9%, hypothyroidism between 3.5 and 25.4%, hypogonadism between 10.2 and 51.1%, and the incidence of retention was between 0.1 and 30%. Unlike other target axes, although the difference was not statistically significant, patients with growing tumors had an increased risk of adrenal axis dysfunction after endoscopic surgery. A retrospective study of 36 patients with acromegaly suggests that hypothalamic–pituitary–adrenal (HPA) axis function may worsen over time in patients with acromegaly, regardless of the type of treatment. In contrast to the deterioration of the HPA axis, thyroid and gonadal function did not change over the same period. The effect of surgery on the HPA axis was considered to be caused by progressive fibrosis and vascular remodeling after surgical intervention [50]. Anshu Buttan et al.’s study on endocrine outcomes after pituitary surgery showed that when the preoperative adrenal function was intact, 4–9% of patients would suffer from postoperative secondary adrenal dysfunction, and 18% of patients might experience early transient adrenal dysfunction. The incidence of new hypothyroidism after surgery was about 3%, and 7% recovered after surgery for nonfunctional tumors. New gonadal dysfunction occurs in 1 to 5% of patients, and 6 to 22% of patients with nonfunctional adenomas recover [51]. Therefore, adrenal dysfunction is a common endocrine outcome after pituitary surgery, which is not parallel to other target axes, which is an important reminder for clinicians’ daily management. The specific reasons and mechanisms need to be further explored, but the impact of surgery on patients’ physical state, including stress and other aspects, may also be a part of it. In contrast to new-onset pituitary dysfunction, the existing preoperatively impaired pituitary function can be relieved by surgery [52]. However, in most cases, pituitary recovery is partial and requires continued hormone replacement therapy after surgery [53]. A complete postoperative endocrine laboratory assessment is usually performed at 6 and 12 weeks postoperatively [54]. Meanwhile, the current guidelines recommend up to 1 year of postoperative endocrinological follow-up in patients with normal pituitary function and lifelong follow-up in patients with abnormal pituitary function or with radiation therapy [55].

## Conclusion

To sum up, although there was no significant statistical difference in our results, in patients with somatotroph tumors after undergoing endoscopic surgery, the risk of dysfunction and pituitary insufficiency tends to increase which needs attention in clinical management, while preoperative thyroid insufficiency, gonadal insufficiency, and hyperprolactinemia will be partially relieved. The biochemical response rate of patients with transsphenoidal endoscopic growth hormone tumor resection may increase as endoscopic surgery matures, but some patients still require follow-up treatment, including gamma-ray therapy or drug therapy. Long-term follow-up and drug replacement therapy are required for newly diagnosed pituitary dysfunction, while the existing preoperative hypophysis can be ameliorated to some extent.

## Supplementary Information

**Additional file 1:** **Supplementary Table 1.** Baseline data of included study.

**Additional file 2:** **Supplementary Table 2.** Information on the literature involving postoperative adrenal axial dysfunction.

## Data Availability

Not applicable.
