# Supplementary material for: The effect of endoscopic transsphenoidal somatotroph tumors resection on pituitary hormones: systematic review and meta-analysis
Source: World J Surg Oncol. 2023 Mar 1;21:71. doi: 10.1186/s12957-023-02958-2 (PMC9976528; doi:10.1186/s12957-023-02958-2)
Supplement: Supplementary file 1 — Additional file 1: Supplementary Table 1. Baseline data of included study. [file 12957_2023_2958_MOESM1_ESM.docx]

| Supplementary table 1. Baseline data of included study. | | | | | | | | | | |  |
| --- | --- | --- | --- | --- | --- | --- | --- | --- | --- | --- | --- |
| Author/Year | National | Center | Study design | No. of patients at baseline | Tumour sizes（mm）/volume(mm3) | Gender（male/female） | Average age，mean | Duration of follow-up, m | Study period | Study quality | Ref. |
| Xuan Gong,2022 | China | Single | Retrospective | 11 | N/A | N/A | N/A | N/A | January 2017 to June 2020 | 9 | [1] |
| Petros Stefanidis,2022 | Greece | Single | Retrospective | 24 | N/A | N/A | N/A | 36 | January 2014 to October 2019 | 9 | [2] |
| Alexander P. Kelly,2022 | America | Single | Retrospective | 2 | 14.00mm | 0/2 | 11.5 | N/A | 2005 to 2020 | 8 | [3] |
| Yi Yuen Wang,2022 | Britain | Multi-center | Retrospective | 81 | N/A | 40/41 | 49 | 44 | 2005 to 2015 | 9 | [4] |
| Jun-Lin Yan，2022 | China（Taiwang） | Single | Retrospective | 83 | N/A | 39/44 | 41.1 | 54.2 | 1999 to 2010 | 9 | [5] |
| Marta Araujo-Castro,2021 | Spain | Single | Retrospective | 40 | N/A | 13/27 | 16.9 | 59.2 | 2008 to 2019 | 9 | [6] |
| Chao Tao,2021 | China | Single | Retrospective | 13 | N/A | N/A | N/A | N/A | March 2018 to June 2019 | 9 | [7] |
| NidanQiao,2021 | China | Single | Emulated trial | 345 | 9mm3 | 196/228 | 41.4 | N/A | 2010 to 2019 | 7 | [8] |
| Tugrul Cem Unal,2021 | Turkey | Single | Retrospective | 73 | N/A | 38/35 | 49.3 | 44.1 | January 2009 to January 2019 | 9 | [9] |
| Juan Luis Gómez-Amador,2021 | Mexico | Single | Retrospective | 13 | 23mm3 | 3/10 | 38.5 | 28.3 | January 2014 to March 2019 | 9 | [10] |
| Chin Taweesomboonyat,2021 | Thailand | Single | Retrospective | 30 | N/A | N/A | N/A | N/A | January 2003 to December 2019 | 9 | [11] |
| M Araujo-Castro ,2021 | Spain | Multi-center | Retrospective | 49 | N/A | 16/33 | 50.4 | N/A | N/A | 9 | [12] |
| Savas Ceylan，2021 | Turkey | Single | Retrospective | 7 | N/A | N/A | N/A | N/A | August 1997 to December 2019 | 8 | [13] |
| Xiaopeng Guo，2021 | China | Single | Prospective | 529 | 16.7mm | 235/294 | 41.5 | 34 | January 2015 to December 2018 | 9 | [14] |
| EnesAkkaya，2021 | Turkey | Single | Retrospective | 75 | N/A | 28/47 | N/A | N/A | 2014 to 2019 | 9 | [15] |
| AbhijitGoyal-Honavar，2021 | India | Single | Retrospective | 203 | 23.4mm | 88/115 | 37.1 | 35.3 | June 2008 to August 2019 | 9 | [16] |
| Yong Zheng,2020 | China | Single | Retrospective | 33 | N/A | 15/18 | 43.24 | N/A | January 2013 to December 2017 | 9 | [17] |
| Tyler Cardinal，2020 | America | Multi-center | Retrospective | 52 | 16.0mm | 16/36 | 50.7 | 24.4 | September 2012 to June 2019 | 9 | [18] |
| Liang Lv，2019 | China | Single | Retrospective | 94 | N/A | 41/53 | N/A | 35 | January 2008 to December 2017 | 9 | [19] |
| Ana M. Castaño-Leon,2019 | Spain | Single | prospective | 16 | N/A | N/A | N/A | N/A | October 12, 1995 to 2017 | 9 | [20] |
| Mohammed J Asha,2019 | Canada | Single | Retrospective | 62 | N/A | N/A | N/A | N/A | January 2000 to June 2016 | 9 | [21] |
| Davide Locatelli,2019 | Italy | Multi-center | Retrospective | 5 | 31.2mm | 5/0 | 16.2 | N/A | N/A | 8 | [22] |
| Mohammad Taghvaei,2018 | Iran | Single | Retrospective | 68 | 17.97mm | 36/32 | 39.35 | 20.97 | September 2013 to December 2016 | 9 | [23] |
| Ihsan Anik,2017 | Turkey | Single | Retrospective | 401 | N/A | 189/212 | 42.09 | 13 to 182 | August 1997 to June 2016 | 9 | [24] |
| Guan Sun,2017 | China | Single | Retrospective | 7 | N/A | N/A | N/A | N/A | September 2013 to December 2015 | 9 | [25] |
| Heping Zhou,2017 | China | Single | Retrospective | 86 | N/A | 45/41 | 42.6 | 27.6 | January 2010 to December 2016 | 9 | [26] |
| ShigetoshiYano，2017 | Japan | Single | Retrospective | 47 | 18.3mm | 12/35 | 46.9 | 32.3 | February 2002 to October 2016 | 9 | [27] |
| Caroline Martins Dos Santos Leopoldo，2017 | Brazil | Single | Retrospective | 23 | N/A | N/A | N/A | N/A | February 2005 to April 2015 | 9 | [28] |
| Jung HeeKim，2017 | Korea | Multi-center | Retrospective | 134 | 20.1mm | 66/68 | 46.1 | at least 6 | January 2009 to March 2016 | 9 | [29] |
| David Netuka，2016 | Czech Republic | Single | Retrospective | 105 | 28.38mm3 | 51/54 | 48.3 | 35 | January 2009 to December 2014 | 9 | [30] |
| OzlemHaliloglu，2016 | Turkey | Single | Retrospective | 103 | N/A | 60/43 | 44.2 | 38 | 2007 to 2014 | 9 | [31] |
| Hussein Fathalla，2015 | Canada | Single | Retrospective | 42 | 36.2mm3 | 21/21 | 43.2 | N/A | 2000 to 2013 | 9 | [32] |
| J Lenzi,2015 | Italy | Single | Retrospective | 22 | N/A | N/A | N/A | N/A | 1996 to 2006 | 8 | [33] |
| Fuyu Wang,2015 | China | Single | Retrospective | 180 | N/A | N/A | N/A | 28.8 | January 2007 to June 2013 | 9 | [34] |
| Tao Zhou，2014 | China | Single | Retrospective | 133 | N/A | 96/37 | 38.3 | 3 to 48 | January 2007 to July 2012 | 9 | [35] |
| Alessandro Paluzzi，2014 | America | Single | Retrospective | 49 | N/A | N/A | N/A | 37.2 | April 2002 to August 2011 | 9 | [36] |
| Yildirum A，2014 | Turkey | Single | Retrospective | 56 | N/A | 17/39 | 42.3 | 18 | June 2009 to September 2013 | 9 | [37] |
| Sauradeep Sarkar，2014 | India | Single | Retrospective | 66 | 22.0mm | 30/33 | 37.6 | 22.38 | January 2005 to April 2013 | 9 | [38] |
| Hiroshi Nishioka,2014 | Japan | Single | Retrospective | 150 | N/A | 77/73 | 47 | 22 | 2011 to 2012 | 9 | [39] |
| Carlos Takahiro Chone,2014 | Brazil | Single | Retrospective | 7 | N/A | N/A | N/A | 15 | January 2009 to December 2012 | 8 | [40] |
| Derya Burcu Hazer，2013 | Turkey | Single | Retrospective | 214 | N/A | 106/108 | 41.9 | N/A | N/A | 9 | [41] |
| Christa C. van Bunderen，2013 | Netherlands | Multi-center | Retrospective | 30 | N/A | 18/12 | 43.6 | 13.2 | 2001 to 2009 | 9 | [42] |
| Robert M. Starke，2013 | America | Single | Retrospective | 72 | 17.6mm | 40/32 | 49.2 | 18.4 | August 2004 to December 2009 | 9 | [43] |
| Adam N Mamelak, 2013 | America | Single | Retrospective | 41 | N/A | N/A | N/A | 36.6 | 2006 to 2011 | 9 | [44] |
| Thomas J Wilson,2013 | America | Single | Retrospective | 14 | 13.4mm | 6/8 | 41.4 | N/A | April 1, 2003 and 2012 | 9 | [45] |
| Shin SS,2013 | America | Single | Retrospective | 53 | 19.4mm | 30/23 | 43.7 | 30 | November 1998 to February 2012 | 9 | [46] |
| Andreja Marić,2012 | Croatia | Single | Retrospective | 21 | N/A | 9/12 | 50.6 | N/A | 2007 to 2010 | 9 | [47] |
| Christoph P Hofstetter,2011 | America | Single | prospective | 33 | N/A | 19/14 | 49.9 | 22.2 | February 2004 and June 2010 | 9 | [48] |
| Yi Yuen Wang，2011 | Britain | Single | Retrospective | 43 | N/A | N/A | 49 | 34 | 2005 to 2010 | 9 | [49] |
| John A Jane Jr ，2011 | America | Single | Retrospective | 60 | N/A | 33/27 | 48 | 21.84 | August 2004 to December 2009 | 9 | [50] |
| Wagenmakers MA，2011 | Netherlands | Single | Retrospective | 40 | 18 mm | 19/21 | 47.4 | 56 | 1998 to 2007 | 9 | [51] |
| Américo Rubens Leite dos Santos,2010 | Brazil | Single | Retrospective | 8 | N/A | N/A | N/A | N/A | January 2005 to September 2008 | 8 | [52] |
| Christoph P Hofstetter，2010 | America | Single | prospective | 24 | N/A | 13/11 | 50.7 | 23.2 | February 2004 to May 2010 | 9 | [53] |
| Jackson A Gondim，2010 | Brazil | Single | Retrospective | 67 | 21.5 mm | 32/35 | 44.8 | 24 | 2000 to 2009 | 9 | [54] |
| Peter G Campbell,2010 | America | Single | Retrospective | 26 | 14.11mm3 | 14/12 | 45.7 | 24.5 | June 2005 to September 2009 | 9 | [55] |
| Abtin Tabaee，2009 | America | Single | prospective | 6 | N/A | N/A | N/A | N/A | January 2004 to May 2006 | 9 | [56] |
| Jackson A. Gondim,2009 | Brazil | Single | Retrospective | 58 | 20.8mm | N/A | N/A | N/A | May 1998 to December 2007 | 9 | [57] |
| Jackson A Gondim,2009 | Brazil | Single | Retrospective | 33 | N/A | 9/24 | 44 | 24 | 2000 to 2005 | 9 | [58] |
| Shigetoshi YANO,2009 | Japan | Single | Retrospective | 31 | N/A | N/A | N/A | N/A | December 2001 to March 2008 | 9 | [59] |
| Jean D'Haens，2009 | Belgium | Single | Retrospective | 13 | N/A | N/A | N/A | N/A | February 1995 to September 2001 | 8 | [60] |
| Amir R Dehdashti,2008 | Canada | Single | Retrospective | 34 | N/A | N/A | N/A | 20 | July 2004 to March 2007 | 9 | [61] |
| Jai-Ho Choe，2008 | Korea | Single | Retrospective | 9 | 13.2mm | 5/4 | 51.4 | N/A | N/A | 9 | [62] |
| Giorgio Frank,2006 | Italy | Single | Retrospective | 16 | N/A | N/A | N/A | at least 6 | May 1998 to February 2005 | 9 | [63] |
| M. S. Kabil,2005 | America | Single | Retrospective | 48 | N/A | N/A | N/A | 38.2 | November 1998 to October 2004, | 9 | [64] |
| A Rudnik,2005 | Poland | Single | Retrospective | 12 | N/A | N/A | N/A | N/A | October 2001 to November 2002 | 9 | [65] |
| Paolo Cappabianca ,2002 | Italy | Single | Retrospective | 36 | N/A | N/A | N/A | 3-48 | January 1997 and July 2001 | 9 | [66] |
| WM Lui，2001 | China（Hong Kong） | Single | Retrospective | 5 | N/A | 0/5 | 50.0 | 14.6 | July 1999 to December 1999 | 8 | [67] |
| P Cappabianca,1999 | Poland | Single | Retrospective | 5 | N/A | 3/2 | 50.4 | N/A | N/A | 8 | [68] |
| A Gamea,1994 | Egypt | Single | Retrospective | 5 | N/A | N/A | N/A | N/A | N/A | 7 | [69] |
| Supplementary table 1. Baseline data of included study. | | | | | | | | | | |  |
| Author/Year | National | Center | Study design | No. of patients at baseline | Tumour sizes（mm）/volume(mm3) | Gender（male/female） | Average age，mean | Duration of follow-up, m | Study period | Study quality | Ref. |
| Xuan Gong,2022 | China | Single | Retrospective | 11 | N/A | N/A | N/A | N/A | January 2017 to June 2020 | 9 | [1] |
| Petros Stefanidis,2022 | Greece | Single | Retrospective | 24 | N/A | N/A | N/A | 36 | January 2014 to October 2019 | 9 | [2] |
| Alexander P. Kelly,2022 | America | Single | Retrospective | 2 | 14.00mm | 0/2 | 11.5 | N/A | 2005 to 2020 | 8 | [3] |
| Yi Yuen Wang,2022 | Britain | Multi-center | Retrospective | 81 | N/A | 40/41 | 49 | 44 | 2005 to 2015 | 9 | [4] |
| Jun-Lin Yan，2022 | China（Taiwang） | Single | Retrospective | 83 | N/A | 39/44 | 41.1 | 54.2 | 1999 to 2010 | 9 | [5] |
| Marta Araujo-Castro,2021 | Spain | Single | Retrospective | 40 | N/A | 13/27 | 16.9 | 59.2 | 2008 to 2019 | 9 | [6] |
| Chao Tao,2021 | China | Single | Retrospective | 13 | N/A | N/A | N/A | N/A | March 2018 to June 2019 | 9 | [7] |
| NidanQiao,2021 | China | Single | Emulated trial | 345 | 9mm3 | 196/228 | 41.4 | N/A | 2010 to 2019 | 7 | [8] |
| Tugrul Cem Unal,2021 | Turkey | Single | Retrospective | 73 | N/A | 38/35 | 49.3 | 44.1 | January 2009 to January 2019 | 9 | [9] |
| Juan Luis Gómez-Amador,2021 | Mexico | Single | Retrospective | 13 | 23mm3 | 3/10 | 38.5 | 28.3 | January 2014 to March 2019 | 9 | [10] |
| Chin Taweesomboonyat,2021 | Thailand | Single | Retrospective | 30 | N/A | N/A | N/A | N/A | January 2003 to December 2019 | 9 | [11] |
| M Araujo-Castro ,2021 | Spain | Multi-center | Retrospective | 49 | N/A | 16/33 | 50.4 | N/A | N/A | 9 | [12] |
| Savas Ceylan，2021 | Turkey | Single | Retrospective | 7 | N/A | N/A | N/A | N/A | August 1997 to December 2019 | 8 | [13] |
| Xiaopeng Guo，2021 | China | Single | Prospective | 529 | 16.7mm | 235/294 | 41.5 | 34 | January 2015 to December 2018 | 9 | [14] |
| EnesAkkaya，2021 | Turkey | Single | Retrospective | 75 | N/A | 28/47 | N/A | N/A | 2014 to 2019 | 9 | [15] |
| AbhijitGoyal-Honavar，2021 | India | Single | Retrospective | 203 | 23.4mm | 88/115 | 37.1 | 35.3 | June 2008 to August 2019 | 9 | [16] |
| Yong Zheng,2020 | China | Single | Retrospective | 33 | N/A | 15/18 | 43.24 | N/A | January 2013 to December 2017 | 9 | [17] |
| Tyler Cardinal，2020 | America | Multi-center | Retrospective | 52 | 16.0mm | 16/36 | 50.7 | 24.4 | September 2012 to June 2019 | 9 | [18] |
| Liang Lv，2019 | China | Single | Retrospective | 94 | N/A | 41/53 | N/A | 35 | January 2008 to December 2017 | 9 | [19] |
| Ana M. Castaño-Leon,2019 | Spain | Single | prospective | 16 | N/A | N/A | N/A | N/A | October 12, 1995 to 2017 | 9 | [20] |
| Mohammed J Asha,2019 | Canada | Single | Retrospective | 62 | N/A | N/A | N/A | N/A | January 2000 to June 2016 | 9 | [21] |
| Davide Locatelli,2019 | Italy | Multi-center | Retrospective | 5 | 31.2mm | 5/0 | 16.2 | N/A | N/A | 8 | [22] |
| Mohammad Taghvaei,2018 | Iran | Single | Retrospective | 68 | 17.97mm | 36/32 | 39.35 | 20.97 | September 2013 to December 2016 | 9 | [23] |
| Ihsan Anik,2017 | Turkey | Single | Retrospective | 401 | N/A | 189/212 | 42.09 | 13 to 182 | August 1997 to June 2016 | 9 | [24] |
| Guan Sun,2017 | China | Single | Retrospective | 7 | N/A | N/A | N/A | N/A | September 2013 to December 2015 | 9 | [25] |
| Heping Zhou,2017 | China | Single | Retrospective | 86 | N/A | 45/41 | 42.6 | 27.6 | January 2010 to December 2016 | 9 | [26] |
| ShigetoshiYano，2017 | Japan | Single | Retrospective | 47 | 18.3mm | 12/35 | 46.9 | 32.3 | February 2002 to October 2016 | 9 | [27] |
| Caroline Martins Dos Santos Leopoldo，2017 | Brazil | Single | Retrospective | 23 | N/A | N/A | N/A | N/A | February 2005 to April 2015 | 9 | [28] |
| Jung HeeKim，2017 | Korea | Multi-center | Retrospective | 134 | 20.1mm | 66/68 | 46.1 | at least 6 | January 2009 to March 2016 | 9 | [29] |
| David Netuka，2016 | Czech Republic | Single | Retrospective | 105 | 28.38mm3 | 51/54 | 48.3 | 35 | January 2009 to December 2014 | 9 | [30] |
| OzlemHaliloglu，2016 | Turkey | Single | Retrospective | 103 | N/A | 60/43 | 44.2 | 38 | 2007 to 2014 | 9 | [31] |
| Hussein Fathalla，2015 | Canada | Single | Retrospective | 42 | 36.2mm3 | 21/21 | 43.2 | N/A | 2000 to 2013 | 9 | [32] |
| J Lenzi,2015 | Italy | Single | Retrospective | 22 | N/A | N/A | N/A | N/A | 1996 to 2006 | 8 | [33] |
| Fuyu Wang,2015 | China | Single | Retrospective | 180 | N/A | N/A | N/A | 28.8 | January 2007 to June 2013 | 9 | [34] |
| Tao Zhou，2014 | China | Single | Retrospective | 133 | N/A | 96/37 | 38.3 | 3 to 48 | January 2007 to July 2012 | 9 | [35] |
| Alessandro Paluzzi，2014 | America | Single | Retrospective | 49 | N/A | N/A | N/A | 37.2 | April 2002 to August 2011 | 9 | [36] |
| Yildirum A，2014 | Turkey | Single | Retrospective | 56 | N/A | 17/39 | 42.3 | 18 | June 2009 to September 2013 | 9 | [37] |
| Sauradeep Sarkar，2014 | India | Single | Retrospective | 66 | 22.0mm | 30/33 | 37.6 | 22.38 | January 2005 to April 2013 | 9 | [38] |
| Hiroshi Nishioka,2014 | Japan | Single | Retrospective | 150 | N/A | 77/73 | 47 | 22 | 2011 to 2012 | 9 | [39] |
| Carlos Takahiro Chone,2014 | Brazil | Single | Retrospective | 7 | N/A | N/A | N/A | 15 | January 2009 to December 2012 | 8 | [40] |
| Derya Burcu Hazer，2013 | Turkey | Single | Retrospective | 214 | N/A | 106/108 | 41.9 | N/A | N/A | 9 | [41] |
| Christa C. van Bunderen，2013 | Netherlands | Multi-center | Retrospective | 30 | N/A | 18/12 | 43.6 | 13.2 | 2001 to 2009 | 9 | [42] |
| Robert M. Starke，2013 | America | Single | Retrospective | 72 | 17.6mm | 40/32 | 49.2 | 18.4 | August 2004 to December 2009 | 9 | [43] |
| Adam N Mamelak, 2013 | America | Single | Retrospective | 41 | N/A | N/A | N/A | 36.6 | 2006 to 2011 | 9 | [44] |
| Thomas J Wilson,2013 | America | Single | Retrospective | 14 | 13.4mm | 6/8 | 41.4 | N/A | April 1, 2003 and 2012 | 9 | [45] |
| Shin SS,2013 | America | Single | Retrospective | 53 | 19.4mm | 30/23 | 43.7 | 30 | November 1998 to February 2012 | 9 | [46] |
| Andreja Marić,2012 | Croatia | Single | Retrospective | 21 | N/A | 9/12 | 50.6 | N/A | 2007 to 2010 | 9 | [47] |
| Christoph P Hofstetter,2011 | America | Single | prospective | 33 | N/A | 19/14 | 49.9 | 22.2 | February 2004 and June 2010 | 9 | [48] |
| Yi Yuen Wang，2011 | Britain | Single | Retrospective | 43 | N/A | N/A | 49 | 34 | 2005 to 2010 | 9 | [49] |
| John A Jane Jr ，2011 | America | Single | Retrospective | 60 | N/A | 33/27 | 48 | 21.84 | August 2004 to December 2009 | 9 | [50] |
| Wagenmakers MA，2011 | Netherlands | Single | Retrospective | 40 | 18 mm | 19/21 | 47.4 | 56 | 1998 to 2007 | 9 | [51] |
| Américo Rubens Leite dos Santos,2010 | Brazil | Single | Retrospective | 8 | N/A | N/A | N/A | N/A | January 2005 to September 2008 | 8 | [52] |
| Christoph P Hofstetter，2010 | America | Single | prospective | 24 | N/A | 13/11 | 50.7 | 23.2 | February 2004 to May 2010 | 9 | [53] |
| Jackson A Gondim，2010 | Brazil | Single | Retrospective | 67 | 21.5 mm | 32/35 | 44.8 | 24 | 2000 to 2009 | 9 | [54] |
| Peter G Campbell,2010 | America | Single | Retrospective | 26 | 14.11mm3 | 14/12 | 45.7 | 24.5 | June 2005 to September 2009 | 9 | [55] |
| Abtin Tabaee，2009 | America | Single | prospective | 6 | N/A | N/A | N/A | N/A | January 2004 to May 2006 | 9 | [56] |
| Jackson A. Gondim,2009 | Brazil | Single | Retrospective | 58 | 20.8mm | N/A | N/A | N/A | May 1998 to December 2007 | 9 | [57] |
| Jackson A Gondim,2009 | Brazil | Single | Retrospective | 33 | N/A | 9/24 | 44 | 24 | 2000 to 2005 | 9 | [58] |
| Shigetoshi YANO,2009 | Japan | Single | Retrospective | 31 | N/A | N/A | N/A | N/A | December 2001 to March 2008 | 9 | [59] |
| Jean D'Haens，2009 | Belgium | Single | Retrospective | 13 | N/A | N/A | N/A | N/A | February 1995 to September 2001 | 8 | [60] |
| Amir R Dehdashti,2008 | Canada | Single | Retrospective | 34 | N/A | N/A | N/A | 20 | July 2004 to March 2007 | 9 | [61] |
| Jai-Ho Choe，2008 | Korea | Single | Retrospective | 9 | 13.2mm | 5/4 | 51.4 | N/A | N/A | 9 | [62] |
| Giorgio Frank,2006 | Italy | Single | Retrospective | 16 | N/A | N/A | N/A | at least 6 | May 1998 to February 2005 | 9 | [63] |
| M. S. Kabil,2005 | America | Single | Retrospective | 48 | N/A | N/A | N/A | 38.2 | November 1998 to October 2004, | 9 | [64] |
| A Rudnik,2005 | Poland | Single | Retrospective | 12 | N/A | N/A | N/A | N/A | October 2001 to November 2002 | 9 | [65] |
| Paolo Cappabianca ,2002 | Italy | Single | Retrospective | 36 | N/A | N/A | N/A | 3-48 | January 1997 and July 2001 | 9 | [66] |
| WM Lui，2001 | China（Hong Kong） | Single | Retrospective | 5 | N/A | 0/5 | 50.0 | 14.6 | July 1999 to December 1999 | 8 | [67] |
| P Cappabianca,1999 | Poland | Single | Retrospective | 5 | N/A | 3/2 | 50.4 | N/A | N/A | 8 | [68] |
| A Gamea,1994 | Egypt | Single | Retrospective | 5 | N/A | N/A | N/A | N/A | N/A | 7 | [69] |

1. Gong, X., et al., *Outcome of Endoscopic Transsphenoidal Surgery for Recurrent or Residual Pituitary Adenomas and Comparison to Non-Recurrent or Residual Cohort by Propensity Score Analysis.* Front Endocrinol (Lausanne), 2022. **13**: p. 837025.

2. Stefanidis, P., et al., *Postoperative complications after endoscope-assisted transsphenoidal surgery for pituitary adenomas: a case series, systematic review, and meta-analysis of the literature.* Hormones (Athens), 2022. **21**(3): p. 487-499.

3. Kelly, A.P., et al., *Pediatric pituitary adenomas are more aggressive, more likely to be hormone producing and are more difficult to cure than adult pituitary adenomas: case series and systematic literature review.* Childs Nerv Syst, 2022. **38**(4): p. 729-738.

4. Wang, Y.Y., et al., *Value of Early Post-Operative Growth Hormone Testing in Predicting Long-Term Remission and Residual Disease after Transsphenoidal Surgery for Acromegaly.* Neuroendocrinology, 2022. **112**(4): p. 345-357.

5. Yan, J.-L., et al., *Surgical Outcome and Evaluation of Strategies in the Management of Growth Hormone-Secreting Pituitary Adenomas After Initial Transsphenoidal Pituitary Adenectomy Failure.* Frontiers in Endocrinology, 2022. **13**.

6. Araujo-Castro, M., et al., *Presurgical somatostatin receptor ligand treatment does not affect tumor consistency in GH-secreting pituitary macroadenomas.* Endocr Connect, 2021. **10**(1): p. 102-109.

7. Tao, C., et al., *Early outcomes of endoscopic endonasal approach pituitary adenomas resection with minimal nasal injury.* Medicine (Baltimore), 2021. **100**(46): p. e27843.

8. Qiao, N., et al., *Comparative effectiveness of endoscopic versus microscopic transsphenoidal surgery for patients with growth hormone secreting pituitary adenoma: An emulated trial.* Clin Neurol Neurosurg, 2021. **207**: p. 106781.

9. Unal, T.C., et al., *A single-center experience of transsphenoidal endoscopic surgery for acromegaly in 73 patients: results and predictive factors for remission.* Br J Neurosurg, 2021: p. 1-6.

10. Gómez-Amador, J.L., et al., *Endoscopic endonasal lateral transellar approach for growth hormone-secreting adenomas with cavernous sinus invasion: Technical note and surgical results.* Neurocirugia (Astur : Engl Ed), 2021. **32**(4): p. 170-177.

11. Taweesomboonyat, C. and T. Oearsakul, *Prognostic Factors of Acromegalic Patients with Growth Hormone-Secreting Pituitary Adenoma After Transsphenoidal Surgery.* World Neurosurg, 2021. **146**: p. e1360-e1366.

12. Araujo-Castro, M., et al., *Multidisciplinary protocol of preoperative and surgical management of patients with pituitary tumors candidates to pituitary surgery.* Ann Endocrinol (Paris), 2021. **82**(1): p. 20-29.

13. Ceylan, S., et al., *An endoscopic endonasal approach to craniopharyngioma via the infrachiasmatic corridor: a single center experience of 84 patients.* Acta Neurochir (Wien), 2021. **163**(8): p. 2253-2268.

14. Guo, X., et al., *Hyperprolactinemia and Hypopituitarism in Acromegaly and Effect of Pituitary Surgery: Long-Term Follow-up on 529 Patients.* Front Endocrinol (Lausanne), 2021. **12**: p. 807054.

15. Akkaya, E., et al., *T2-weighted magnetic resonance imaging as a novel predictor of surgical remission in newly diagnosed pituitary macroadenomas presenting as acromegaly.* J Clin Neurosci, 2021. **90**: p. 105-111.

16. Goyal-Honavar, A., et al., *Impact of Experience on Outcomes After Endoscopic Transsphenoidal Surgery for Acromegaly.* World Neurosurg, 2021. **151**: p. e1007-e1015.

17. Zheng, Y., et al., *Surgical management of growth hormone-secreting pituitary adenomas: A retrospective analysis of 33 patients.* Medicine (Baltimore), 2020. **99**(19): p. e19855.

18. Cardinal, T., et al., *Impact of tumor characteristics and pre- and postoperative hormone levels on hormonal remission following endoscopic transsphenoidal surgery in patients with acromegaly.* Neurosurg Focus, 2020. **48**(6): p. E10.

19. Lv, L., et al., *Mammosomatotroph and mixed somatotroph-lactotroph adenoma in acromegaly: a retrospective study with long-term follow-up.* Endocrine, 2019. **66**(2): p. 310-318.

20. Castaño-Leon, A.M., et al., *Endoscopic Transnasal Trans-Sphenoidal Approach for Pituitary Adenomas: A Comparison to the Microscopic Approach Cohort by Propensity Score Analysis.* Neurosurgery, 2020. **86**(3): p. 348-356.

21. Asha, M.J., et al., *Long-term outcomes of transsphenoidal surgery for management of growth hormone-secreting adenomas: single-center results.* J Neurosurg, 2019: p. 1-11.

22. Locatelli, D., et al., *Transsphenoidal surgery for pituitary adenomas in pediatric patients: a multicentric retrospective study.* Childs Nerv Syst, 2019. **35**(11): p. 2119-2126.

23. Taghvaei, M., et al., *Endoscopic Endonasal Approach to the Growth Hormone-Secreting Pituitary Adenomas: Endocrinologic Outcome in 68 Patients.* World Neurosurg, 2018. **117**: p. e259-e268.

24. Anik, I., et al., *Endoscopic Transsphenoidal Approach for Acromegaly with Remission Rates in 401 Patients: 2010 Consensus Criteria.* World Neurosurg, 2017. **108**: p. 278-290.

25. Sun, G., et al., *Binostril endoscopic transsphenoidal neurosurgery for pituitary adenomas: experience with 42 patients.* Oncotarget, 2017. **8**(40): p. 69020-69024.

26. Zhou, H., et al., *Endoscopic therapy and curative effect in pituitary adenoma patients complicated by acromegalic cardiomyopathy.* Neurosurg Rev, 2018. **41**(3): p. 869-875.

27. Yano, S., T. Hide, and N. Shinojima, *Efficacy and Complications of Endoscopic Skull Base Surgery for Giant Pituitary Adenomas.* World Neurosurg, 2017. **99**: p. 533-542.

28. Leopoldo, C., et al., *Long term follow-up of growth hormone-secreting pituitary adenomas submitted to endoscopic endonasal surgery.* Arq Neuropsiquiatr, 2017. **75**(5): p. 301-306.

29. Kim, J.H., et al., *Outcome of Endoscopic Transsphenoidal Surgery for Acromegaly.* World Neurosurg, 2017. **104**: p. 272-278.

30. Netuka, D., et al., *Intraoperative Magnetic Resonance Imaging During Endoscopic Transsphenoidal Surgery of Growth Hormone-Secreting Pituitary Adenomas.* World Neurosurg, 2016. **91**: p. 490-6.

31. Haliloglu, O., et al., *Multidisciplinary Approach for Acromegaly: A Single Tertiary Center's Experience.* World Neurosurg, 2016. **88**: p. 270-276.

32. Fathalla, H., et al., *Endoscopic versus microscopic approach for surgical treatment of acromegaly.* Neurosurg Rev, 2015. **38**(3): p. 541-8; discussion 548-9.

33. Lenzi, J., et al., *Evaluation of trans-sphenoidal surgery in pituitary GH-secreting micro- and macroadenomas: a comparison between microsurgical and endoscopic approach.* J Neurosurg Sci, 2015. **59**(1): p. 11-8.

34. Wang, F., et al., *Endoscopic endonasal transsphenoidal surgery of 1,166 pituitary adenomas.* Surg Endosc, 2015. **29**(6): p. 1270-80.

35. Zhou, T., et al., *Outcome of endoscopic transsphenoidal surgery in combination with somatostatin analogues in patients with growth hormone producing pituitary adenoma.* J Korean Neurosurg Soc, 2014. **56**(5): p. 405-9.

36. Paluzzi, A., et al., *Endoscopic endonasal approach for pituitary adenomas: a series of 555 patients.* Pituitary, 2014. **17**(4): p. 307-19.

37. Yildirim, A.E., et al., *Endoscopic endonasal transsphenoidal treatment for acromegaly: 2010 consensus criteria for remission and predictors of outcomes.* Turk Neurosurg, 2014. **24**(6): p. 906-12.

38. Sarkar, S., et al., *Endocrinological outcomes following endoscopic and microscopic transsphenoidal surgery in 113 patients with acromegaly.* Clin Neurol Neurosurg, 2014. **126**: p. 190-5.

39. Nishioka, H., et al., *Aggressive transsphenoidal resection of tumors invading the cavernous sinus in patients with acromegaly: predictive factors, strategies, and outcomes.* J Neurosurg, 2014. **121**(3): p. 505-10.

40. Chone, C.T., et al., *Endoscopic endonasal transsphenoidal resection of pituitary adenomas: preliminary evaluation of consecutive cases.* Braz J Otorhinolaryngol, 2014. **80**(2): p. 146-51.

41. Hazer, D.B., et al., *Treatment of acromegaly by endoscopic transsphenoidal surgery: surgical experience in 214 cases and cure rates according to current consensus criteria.* J Neurosurg, 2013. **119**(6): p. 1467-77.

42. van Bunderen, C.C., et al., *Predictors of endoscopic transsphenoidal surgery outcome in acromegaly: patient and tumor characteristics evaluated by magnetic resonance imaging.* Pituitary, 2013. **16**(2): p. 158-67.

43. Starke, R.M., et al., *Endoscopic vs microsurgical transsphenoidal surgery for acromegaly: outcomes in a concurrent series of patients using modern criteria for remission.* J Clin Endocrinol Metab, 2013. **98**(8): p. 3190-8.

44. Mamelak, A.N., et al., *Single-surgeon fully endoscopic endonasal transsphenoidal surgery: outcomes in three-hundred consecutive cases.* Pituitary, 2013. **16**(3): p. 393-401.

45. Wilson, T.J., et al., *Repeat endoscopic transsphenoidal surgery for acromegaly: remission and complications.* Pituitary, 2013. **16**(4): p. 459-64.

46. Shin, S.S., et al., *Endoscopic endonasal approach for growth hormone secreting pituitary adenomas: outcomes in 53 patients using 2010 consensus criteria for remission.* Pituitary, 2013. **16**(4): p. 435-44.

47. Marić, A., et al., *Endocrinological outcomes of pure endoscopic transsphenoidal surgery: a Croatian Referral Pituitary Center experience.* Croat Med J, 2012. **53**(3): p. 224-33.

48. Hofstetter, C.P., et al., *Endoscopic endonasal transsphenoidal surgery for functional pituitary adenomas.* Neurosurg Focus, 2011. **30**(4): p. E10.

49. Wang, Y.Y., et al., *Acromegaly surgery in Manchester revisited--the impact of reducing surgeon numbers and the 2010 consensus guidelines for disease remission.* Clin Endocrinol (Oxf), 2012. **76**(3): p. 399-406.

50. Jane, J.A., Jr., et al., *Endoscopic transsphenoidal surgery for acromegaly: remission using modern criteria, complications, and predictors of outcome.* J Clin Endocrinol Metab, 2011. **96**(9): p. 2732-40.

51. Wagenmakers, M.A., et al., *Results of endoscopic transsphenoidal pituitary surgery in 40 patients with a growth hormone-secreting macroadenoma.* Acta Neurochir (Wien), 2011. **153**(7): p. 1391-9.

52. Santos, A.R., et al., *Endoscopic endonasal transsphenoidal approach for pituitary adenomas: technical aspects and report of casuistic.* Arq Neuropsiquiatr, 2010. **68**(4): p. 608-12.

53. Hofstetter, C.P., et al., *Endoscopic endonasal transsphenoidal surgery for growth hormone-secreting pituitary adenomas.* Neurosurg Focus, 2010. **29**(4): p. E6.

54. Gondim, J.A., et al., *Pure endoscopic transsphenoidal surgery for treatment of acromegaly: results of 67 cases treated in a pituitary center.* Neurosurg Focus, 2010. **29**(4): p. E7.

55. Campbell, P.G., et al., *Outcomes after a purely endoscopic transsphenoidal resection of growth hormone-secreting pituitary adenomas.* Neurosurg Focus, 2010. **29**(4): p. E5.

56. Tabaee, A., et al., *Predictors of short-term outcomes following endoscopic pituitary surgery.* Clin Neurol Neurosurg, 2009. **111**(2): p. 119-22.

57. Gondim, J.A., et al., *Endoscopic endonasal transsphenoidal surgery: surgical results of 228 pituitary adenomas treated in a pituitary center.* Pituitary, 2010. **13**(1): p. 68-77.

58. Gondim, J.A., et al., *Outcome of surgical intrasellar growth hormone tumor performed by a pituitary specialist surgeon in a developing country.* Surg Neurol, 2009. **72**(1): p. 15-9; discussion 19.

59. Yano, S., et al., *Endoscopic endonasal transsphenoidal approach through the bilateral nostrils for pituitary adenomas.* Neurol Med Chir (Tokyo), 2009. **49**(1): p. 1-7.

60. D'Haens, J., et al., *Fully endoscopic transsphenoidal surgery for functioning pituitary adenomas: a retrospective comparison with traditional transsphenoidal microsurgery in the same institution.* Surg Neurol, 2009. **72**(4): p. 336-40.

61. Dehdashti, A.R., et al., *Pure endoscopic endonasal approach for pituitary adenomas: early surgical results in 200 patients and comparison with previous microsurgical series.* Neurosurgery, 2008. **62**(5): p. 1006-15; discussion 1015-7.

62. Choe, J.H., et al., *Endocrine outcome of endoscopic endonasal transsphenoidal surgery in functioning pituitary adenomas.* J Korean Neurosurg Soc, 2008. **44**(3): p. 151-5.

63. Frank, G. and E. Pasquini, *Endoscopic endonasal cavernous sinus surgery, with special reference to pituitary adenomas.* Front Horm Res, 2006. **34**: p. 64-82.

64. Kabil, M.S., J.B. Eby, and H.K. Shahinian, *Fully endoscopic endonasal vs. transseptal transsphenoidal pituitary surgery.* Minim Invasive Neurosurg, 2005. **48**(6): p. 348-54.

65. Rudnik, A., et al., *Endoscopic transnasal transsphenoidal treatment of pathology of the sellar region.* Minim Invasive Neurosurg, 2005. **48**(2): p. 101-7.

66. Cappabianca, P., et al., *Surgical complications associated with the endoscopic endonasal transsphenoidal approach for pituitary adenomas.* J Neurosurg, 2002. **97**(2): p. 293-8.

67. Lui, W.M., et al., *Endonasal endoscopic removal of growth-hormone-secreting pituitary adenomas.* Hong Kong Med J, 2001. **7**(2): p. 189-92.

68. Cappabianca, P., et al., *Endoscopic endonasal transsphenoidal approach: an additional reason in support of surgery in the management of pituitary lesions.* Skull Base Surg, 1999. **9**(2): p. 109-17.

69. Gamea, A., M. Fathi, and A. el-Guindy, *The use of the rigid endoscope in trans-sphenoidal pituitary surgery.* J Laryngol Otol, 1994. **108**(1): p. 19-22.
