# Supplementary material for: The effect of endoscopic transsphenoidal somatotroph tumors resection on pituitary hormones: systematic review and meta-analysis
Source: World J Surg Oncol. 2023 Mar 1;21:71. doi: 10.1186/s12957-023-02958-2 (PMC9976528; doi:10.1186/s12957-023-02958-2)
Supplement: Supplementary file 2 — Additional file 2: Supplementary Table 2. Information on the literature involving postoperative adrenal axial dysfunction. [file 12957_2023_2958_MOESM2_ESM.docx]

| Author | Patient numbers（Total） | Events  （adrenal insufficiency） | Country | Published year |
| --- | --- | --- | --- | --- |
| Xiaopeng Guo | 529 | 25（4.7%） | China | 2021 |
| AbhijitGoyal-Honavar | 188 | 31（16.5%） | India | 2021 |
| John A Jane Jr | 58 | 4（6.9%） | America | 2011 |
| WM Lui | 5 | 1（20%） | China | 2001 |
| Tugrul Cem Unal | 73 | 3（4.1%） | Turkey | 2021 |
| Ihsan Anik | 401 | 6（1.5%） | Turkey | 2017 |
| Margreet Albertina EM Wagenmakers， | 40 | 3（7.5%） | Netherlands | 2011 |
| Robert M. Starke | 72 | 13（18.1%） | America | 2013 |
| SauradeepSarkar | 66 | 4（6.1%） | India | 2014 |
| Jung HeeKim | 134 | 9（6.7%） | Korea | 2017 |
